# Mandibular Brown Tumor of Secondary Hyperparathyroidism Requiring Extensive Resection: A Forgotten Entity in the Developed World?

**DOI:** 10.1155/2015/567543

**Published:** 2015-08-19

**Authors:** Mohammed Qaisi, Matthew Loeb, Lindsay Montague, Ron Caloss

**Affiliations:** ^1^Oral-Head & Neck Oncology/Microvascular Surgery, Department of Oral-Maxillofacial Surgery & Pathology and Department of Otolaryngology, Cancer Institute, University of Mississippi Medical Center, 2500 North State Street, Jackson, MS 39216, USA; ^2^School of Dentistry, University of Mississippi Medical Center, 2500 North State Street, Jackson, MS 39216, USA; ^3^Oral & Maxillofacial Pathology, Department of Oral-Maxillofacial Surgery & Pathology, University of Mississippi Medical Center, 2500 North State Street, Jackson, MS 39216, USA; ^4^Oral & Maxillofacial Surgery, Department of Oral-Maxillofacial Surgery & Pathology, University of Mississippi Medical Center, 2500 North State Street, Jackson, MS 39216, USA

## Abstract

Brown tumor of hyperparathyroidism (BTHPT) is rare in the United States and not frequently seen in clinical practice. This is likely because early diagnosis and prompt treatment of this disease process prevent the progression and development of BTHPT. Conversely, BTHPT is more common in underdeveloped countries where fewer patients have access to health care and hyperparathyroidism (HPT) goes untreated. It has been reported that the incidence of BTHPT in underdeveloped countries can be as high as 58 to 69 percent in patients with primary HPT. We present a case report of a patient in the United States with a large mandibular BTHPT requiring an extensive resection in the setting of secondary HPT. Despite being rare in this country, it is important for nephrologists, primary care physicians, and oral health care providers to be able to recognize this entity, so that intervention may be rendered early.

## 1. Introduction

In a healthy individual, the parathyroid glands and kidneys play an important role in the homeostasis and regulation of serum calcium (Ca) and phosphorus (P) levels. Parathyroid hormone (PTH) is released in response to decreased serum Ca and functions to increase this by causing an efflux of Ca from the bony skeleton and increased reabsorption by the kidneys. PTH also leads to increased release of vitamin D from the kidneys, which in turn causes increased Ca absorption from the gastrointestinal tract. Conversely, PTH leads to decreased P levels due to increased excretion by the kidneys [1].

Hyperparathyroidism (HPT) is a disorder characterized by an excessive amount of parathyroid hormone secretion by the parathyroid glands. Depending on the cause of this PTH production, HPT can be characterized into primary, secondary, and tertiary forms [2, 3].

Primary HPT occurs when one or more parathyroid glands secrete an excessive amount of PTH, as in the case of a parathyroid adenoma; secondary HPT results when increased secretion of PTH is a response to lowered ionized calcium, typically as a result of renal disease [4]. In tertiary HPT, secretion of PTH occurs as a result of long-standing chronic renal disease eventually leading to overactive parathyroid glands that become independent of the underlying disease. Hence, tertiary HPT is not corrected when patients receive a renal transplant that corrects the underlying renal etiology [5].

When any of these forms of HPT are not well controlled, BTHPT may result. These nonneoplastic lesions present late in untreated disease and are more commonly seen in underdeveloped countries due to the lack of access to care; they are infrequently seen in developed countries [6]. These lesions appear identical to central reparative giant cell granulomas histologically and result because of the abnormal calcium homeostasis in HPT [2]. Treatment for these lesions is often directed at the management of the underlying HPT, which frequently results in regression and resolution of these lesions without surgical intervention. However, surgical treatment may be required in refractory cases or in large symptomatic lesions [7].

## 2. Case Report

**Figure 1:**
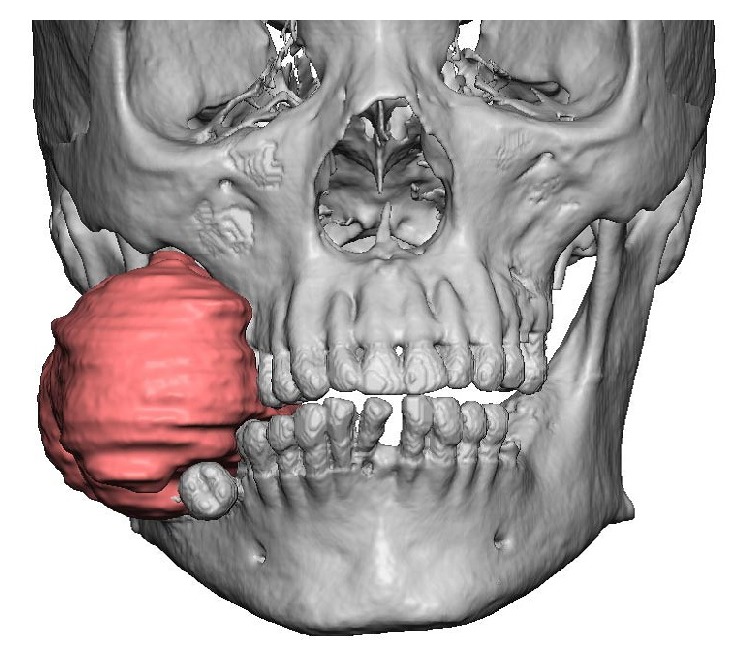
Computer simulation of patient with a large swelling on the lower right side of his face.

**Figure 2:**
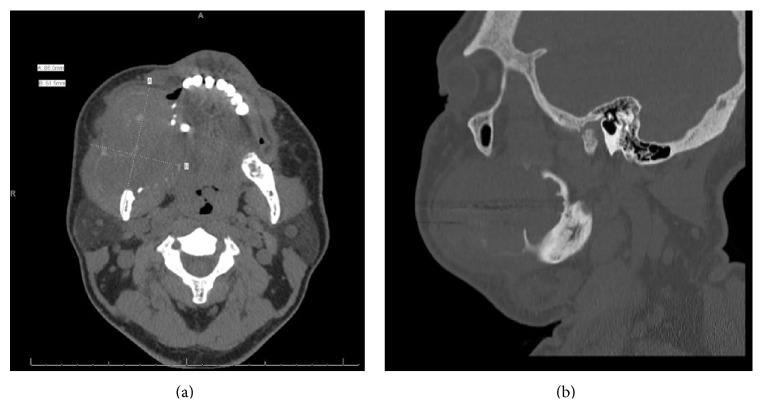
(a) Axial CT image. (b) CT scan, sagittal view.

A 43-year-old African American male presented to the Division of Oral Oncology at the University of Mississippi Cancer Institute with the chief complaint of swelling in his right mandible that had been present for approximately one year (Figure 1). Oral exam revealed a 5.0 × 7.0 cm bulbous mass arising from the mandible and obliterating the right side of the oral cavity. The mass was ulcerated due to occluding with the opposing dentition. The patient denied pain but reported difficulty in eating. A computed tomography (CT) scan (Figures 2(a) and 2(b)) showed an expansile osseous lesion, involving the entire height of the mandible and extending from the right first premolar all the way posteriorly to involve the ramus and coronoid process. This lesion caused severe bony destruction of the mandible, eroding through both buccal and lingual cortices and filling the right half of the oral cavity.

The patient's past medical history was significant for end stage renal disease (ESRD). He was on hemodialysis and was followed by a nephrologist. He had secondary HPT which was not well controlled, with a PTH level of 1,818 pg/mL at the time of presentation (normal range (NR) 11 to 77 pg/mL). This was thought to be likely due to noncompliance with his medications and due to refractory disease. The remainder of his labs was as follows: Ca 8.4 (NR 9–10.5 mg/dL), P 5.3 (NR 3.0–4.5 mg/dL), blood urea nitroge 28 (NR 8–20 mg/dL), and creatinine 7.17 (NR 0.7–1.3 mg/dL).

Based on the past medical history, this lesion was highly suspicious for BTHPT. However, given the rarity of brown tumors and the clinical appearance and size of this lesion, other osseous and odontogenic tumors could not be excluded. An incisional biopsy was performed and histologic examination confirmed the diagnosis of BTHPT.

**Figure 3:**
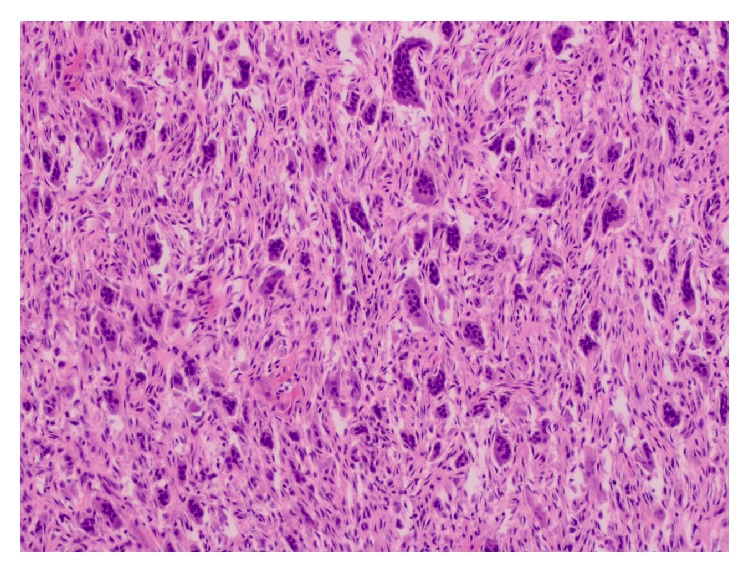
Photomicrograph of the mandibular brown tumor showing numerous multinucleated giant cells dispersed in a highly cellular stroma (H&E, magnification ×200).

**Figure 4:**
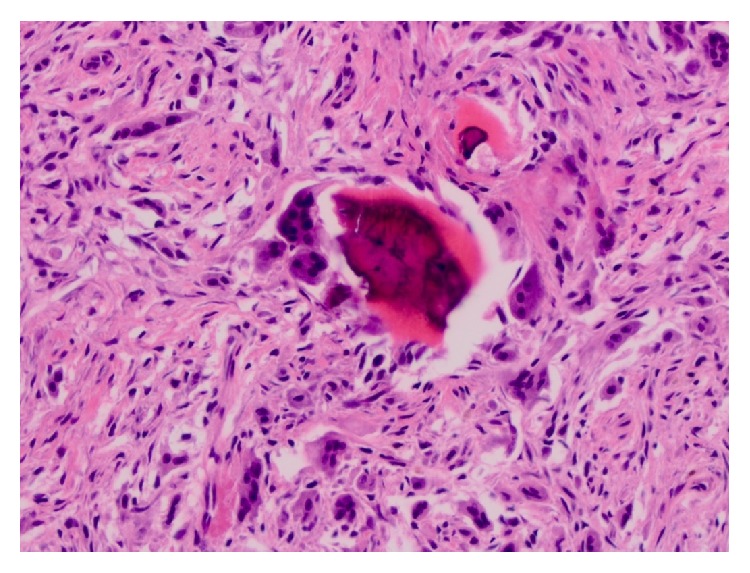
Photomicrograph showing brown tumor of hyperparathyroidism. Multinucleated giant cells surrounding a spicule of bone are visible amidst a proliferative fibroblastic stroma (H&E, magnification ×400).

**Figure 5:**
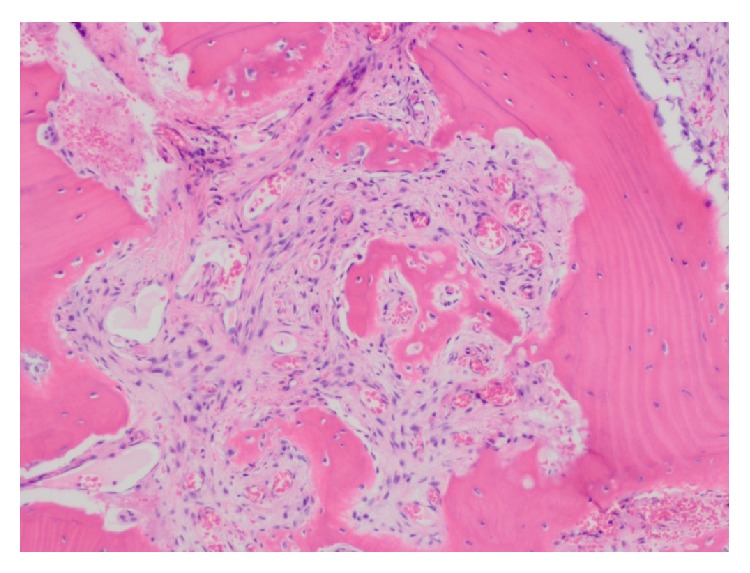
Photomicrograph showing osteoclastic bone resorption and fibrosis in secondary hyperparathyroidism (H&E, magnification ×200).

Microscopically, the lesion was composed of a highly cellular proliferation of bland spindle-shaped fibroblastic cells with numerous multinucleated giant cells (Figure 3) and spicules of woven bone. The osteoclastic giant cells were scattered throughout the cellular stroma and surrounded small spicules of bone (Figure 4). Small blood vessels, extravasated red blood cells, and focal deposits of hemosiderin were seen throughout the lesion, especially toward the periphery. The native bone showed evidence of intense bone resorption secondary to osteoclast hyperactivity and marrow fibrosis, consistent with secondary hyperparathyroidism (Figure 5). The histologic appearance of the lesion was akin to central giant cell reparative granuloma.

**Figure 6:**
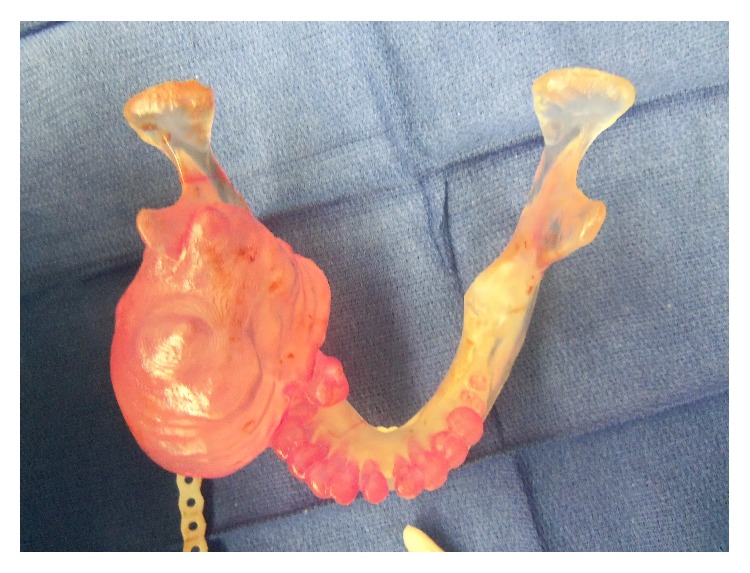
Medical model demonstrating the extent of the expansion of this lesion.

**Figure 7 fig7:**
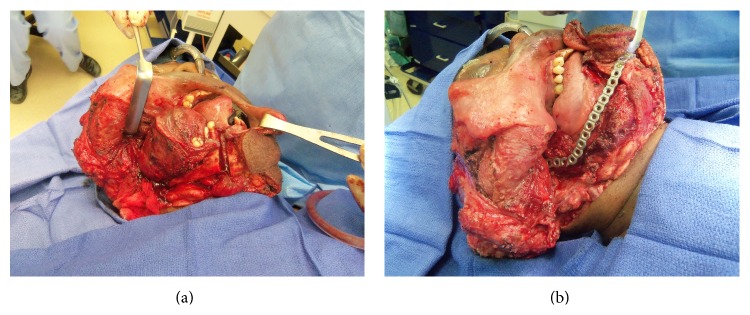
(a) Access to large brown tumor via lip split mandibulotomy approach. (b) Reconstruction bar placement.

**Figure 8:**
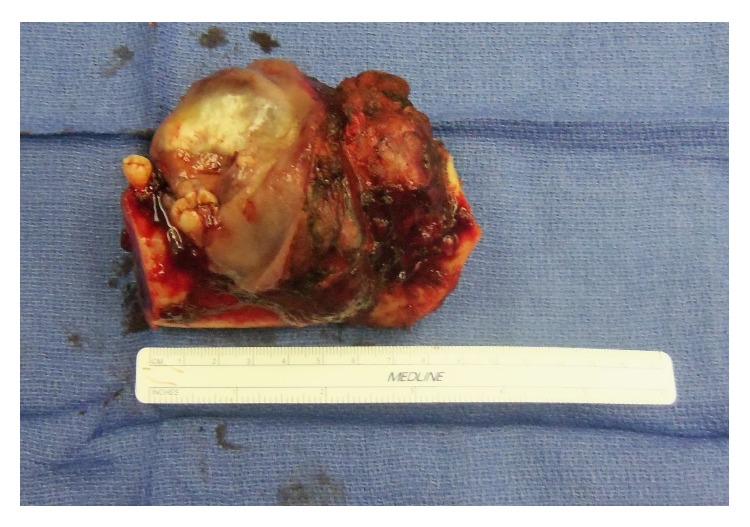
Final surgical specimen.

**Figure 9:**
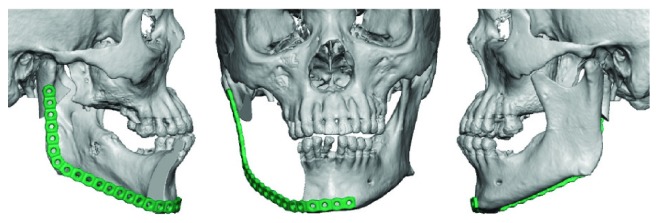
Simulated surgical procedure.

A discussion was held with the patient and treating nephrologist. Given the extent of the tumor and the uncontrolled underlying disease, it was decided that surgical intervention was the best option. Segmental resection with minimal margins was performed because marginal resection or enucleation procedures were deemed not feasible due to the limited amount of bone. This was done via a transcervical lip splitting approach due to limited access from the extensive size of the tumor (Figures 6 [Fig fig7]–8 and supplemental video in Supplementary Material available online at http://dx.doi.org/10.1155/2015/567543). A microvascular free tissue transfer was not performed at that time because the treating nephrologist did not feel the patient could tolerate a lengthy surgery. Virtual surgical planning was utilized to help plan the mandibular resection as well as the reconstruction bar placement (Figure 9). Postoperatively, the patient's hospital course was uneventful. He was followed up by the inpatient nephrology team and continued to receive dialysis while he remained in the hospital.

The patient was followed up for several weeks after surgery, and initial healing was within normal limits. Unfortunately, the patient was noncompliant with long term follow-up despite very extensive efforts to get him rescheduled. This noncompliance may have potentially contributed to the initial presentation of the tumor.

## 3. Discussion

BTHPT is rare in the United States and not frequently seen in clinical practice. Conversely, BTHPT is more common in underdeveloped countries where fewer patients have access to health care and HPT goes untreated. A review of the literature over the last 10 years identified a total of 87 patients in 57 reports with BTHPT of the facial region. Analysis of this data is provided in Table 1. Only 5 of those cases occurred in the United States [8–12]. Of those, four had primary HPT, and one had secondary disease, as our patient did. The highest number of BTHPTs was reported from Mexico, India, and Brazil, respectively. Of the total 87 patients identified with BTHPT, 64 were females and 23 were males (2.8 : 1). The mean age was 42 years (11 to 83). Two-thirds (66.7%) were comprised of primary HPT, while secondary HPT made up 27.6% of cases; tertiary HPT made the remaining 5.7%. With regard to site of occurrence, 48% occurred in the mandible, 38% occurred in the maxilla, and 14% involved both jaws.

BTHPT occurs late in the setting of HPT and is considered as a sign of poorly controlled disease. The prevalence of BTHPT is low, with a reported frequency of 3% in primary HPT and 2% in secondary HPT [13]. Brown tumors occur due to abnormal calcium homeostasis in the setting of uncontrolled parathyroid disease, resulting in significant bone demineralization. Long-standing brown tumors, also known as osteitis fibrosa cystica, often undergo central degeneration and exhibit fibrous marrow replacement, causing a cyst-like radiographic appearance [13]. Histologically, these lesions are identical to central reparative giant cell granulomas, consisting of a spindle cell stroma and numerous multinucleated osteoclast-like giant cells. This lesion is referred to as “brown tumor” due to the reddish brown color of the tissue specimen, secondary to extravasated red blood cells and hemosiderin deposition [7, 14]. Because it is difficult to distinguish histologically between BTHPT and other giant cell lesions, the clinical diagnosis is made based on serum chemistries and the presence or absence of HPT [15].

Primary HPT is the third most common endocrine disorder. The prevalence is reported at 1 per 1000 in the United States [5]. The most common cause of primary HPT is single gland adenoma, which represents around 90% of cases [2, 5, 7, 14]. In most cases, primary HPT is diagnosed incidentally by hypercalcemia and patients are often asymptomatic [16]. However, less frequently, patients with primary HPT will display signs and symptoms of the disease. Symptoms are often related to chronic hypercalcemia and can include nephrolithiasis, muscle weakness, osteoporosis, and psychiatric symptoms [5, 14]. Secondary HPT can be caused by either a vitamin D deficiency or chronic kidney disease, both of which lead to hypocalcemia and thus stimulation of the parathyroid glands. Unlike primary HPT, secondary HPT is typically associated with serum hypocalcemia and hyperphosphatemia [5, 7]. Patients with chronic kidney disease display renal osteodystrophy, which can result in bone loss, bone pain, and fractures [17].

The treatment of BTHPT varies from case to case. However, the treatment of choice related to primary HPT is most commonly to remove the offending parathyroid gland [4]. Many authors report that, after parathyroidectomy, tumor regression and healing will occur [18–22]. The most common approach to identifying the offending parathyroid gland is imaging using technetium (99mTc) scan [23]. In contrast, the treatment of secondary HPT revolves around controlling the underlying ESRD or vitamin D deficiency rather than removal of the parathyroid glands [24]. Medical management includes hemodialysis, low phosphate renal diet, treatment with calcimimetic drugs such as Cinacalcet, and vitamin D derivatives [1]. Renal transplantation, another option for patients with ESRD, can successfully reestablish normal kidney function [2]. Medical treatment has been shown to be sufficient in helping resolve these lesions, although the process is usually slow [25–27]. Conversely, in cases of refractory disease, parathyroidectomy may still be required, in which case total parathyroidectomy is performed with removal of all four glands. Calcium supplementation for life is required in these patients [1, 5, 27].

Although removal of the parathyroid gland in HPT has been shown to lead to regression of the brown tumor and osseous remodeling, the patient's age can be a factor in the rate of regression [16, 20]. When regression is slow, surgical removal of the tumor following parathyroidectomy has been shown to expedite the healing process [24, 28]. Some have also reported tumors failing to regress and continuing to grow even after HPT has been resolved, thus requiring surgical intervention [29, 30]. Others have reported success with the use of intralesional steroid therapy to promote tumor regression; in the same manner, this method has been used for central reparative giant cell granulomas [12, 31].

Table 1 summarizes the methods of treatment for the 87 cases of maxillofacial BTHPT identified in the last 10 years, where data was available. Over 50% of patients were treated with parathyroidectomy or medical management alone. The rest had a combination of treatment modalities, although the extent of surgical curettages and excisions were difficult to assess from reviewing these reports.

In our case, surgical excision was felt to be warranted due to the refractory nature of the secondary HPT and poor compliance with medical treatment. Furthermore, the tumor was large and compromised normal function and esthetics. Patients with ESRD can have recurrence of their tumors if the renal disease is not well controlled postoperatively [32]. Our patient is at risk of developing additional brown tumors due to his poor compliance with medical therapy and the lack of surgical follow-up. We do realize that this lack of surgical follow-up is a limitation of this case report.

## 4. Conclusion

Brown tumors are rare in developed countries, with only 5 cases reported in the United States over the last 10 years at the time of this writing. These occur more frequently in developing countries because they are usually the result of uncontrolled and untreated HPT, perhaps due to lack of access to care. Regression of BTHPT can often be achieved with medical therapy or performing parathyroidectomy in primary HPT, especially if the brown tumor is small. Larger lesions, such as the one described here, usually require some sort of surgical intervention. Additionally, microvascular reconstruction may not be possible in patients with ESRD with multiple comorbidities due to the risks associated with longer anesthesia.

## Supplementary Material

This video shows the surgical technique that was utilized for resection of this tumor. It also illustrates the size of the tumor and soft tissue involvement, necessitating a lip split mandibulotomy approach. Note that the tumor involves the mandibular ramus and encroaches on the condylar region as shown in the accompanying CT images.

## Figures and Tables

**Table 1:** Summary of cases of brown tumor of hyperparathyroidism in the literature.

Author	Year	Country	Patient age	Patient gender	Tumor location	Type of HPT	Treatment	Lesion size (CM)
Chowdhury et al. [33]	2013	Brazil	20	F	MAX & MAND	1° HPT	PTE	3 × 3

Pawlak et al. [34]	2013	Poland	32	F	MAND	1° HPT	PTE + EX BT	na
			57	F	MAND	1° HPT	PTE + EX BT	na
			42	F	MAX	1° HPT	PTE + EX BT	na
			66	F	MAX & MAND	1° HPT	PTE + EX BT	na
			32	M	MAX & MAND	1° HPT	PTE + EX BT	na

de Ávila et al. [35]	2012	Brazil	21	M	MAND	3° HPT	PTE + CUR BT	na

Pace and Crosher [3]	2010	UK	27	F	MAX & MAND	2° HPT	MED MGMT	na

Angadi et al. [36]	2010	India	38	M	MAND	1° HPT	PTE + EX BT	6 × 4

Benhammou et al. [37]	2009	France	23	F	MAX & MAND	1° HPT	PTE	na

Selvi et al. [38]	2009	Turkey	19	M	MAX & MAND	3° HPT	MED MGMT	3.45 × 5.45 × 3.46

Karabekmez et al. [39]	2008	Turkey	11	M	MAX & MAND	2° HPT	na	15 × 20

Tarrass et al. [40]	2008	Morocco	18	M	MAND	2° HPT	PTE	3 × 3

Jebasingh et al. [41]	2008	India	68	M	MAX	1° HPT	PTE	na

Dinkar et al. [42]	2007	India	36	F	MAND	1° HPT	na	na

Desigan et al. [43]	2007	UK	27	F	MAND	1° HPT	PTE	2 × 2

Pinto et al. [31]	2006	Brazil	12	F	MAND	3° HPT	ICCT	na

Prado et al. [27]	2006	Brazil	45	F	MAND	2° HPT	PTE	2 × 2

Triantafillidou et al. [7]	2006	Greece	76	F	MAX & MAND	1° HPT	PTE	na
			71	M	MAND	1° HPT	PTE	na
			21	F	MAND	2° HPT	CUR BT + MED MGMT	na
			70	F	MAND	2° HPT	CUR BT + MED MGMT	na
			68	F	MAND	2° HPT	CUR BT + MED MGMT	na

Grulois et al. [44]	2005	Belgium	57	F	MAND	1° HPT	na	na

Fernández-Sanromán et al. [45]	2005	Spain	16	F	MAND	1° HPT	PTE + CUR BT	na

Jović et al. [46]	2004	Serbia	25	M	MAX	2° HPT	PTE + EX BT	na

Sumer et al. [32]	2004	Saudi Arabia	41	F	MAND	2° HPT	PTE	3.5 × 3

Emin et al. [47]	2004	Turkey	62	F	MAND	1° HPT	PTE + EX BT	7 × 5 × 3

Suarez-Cunqueiro et al. [18]	2004	Germany	26	M	MAND	1° HPT	PTE	na

Gangidi et al. [19]	2012	UK	83	F	MAND	1° HPT	PTE	na

Placed et al. [48]	2010	Spain	33	F	MAX	2° HPT	na	na

Pinto et al. [22]	2010	Brazil	37	F	MAX	2° HPT	PTE	na

Pérez-Guillermo et al. [49]	2006	Spain	61	M	MAX	2° HPT	PTE + EX BT	3 × 2

Daniels [16]	2004	Saudi Arabia	25	F	MAX	1° HPT	PTE + CUR BT	na

Reséndiz-Colosia et al. [20]	2008	Mexico	51	F	MAX	1° HPT	PTE	5
			27	F	MAX	1° HPT	PTE	4
			62	M	MAX	1° HPT	PTE	4
			77	F	MAX	1° HPT	PTE	3
			48	M	MAX	1° HPT	PTE	4
			57	F	MAX	1° HPT	PTE	4
			64	F	MAX	1° HPT	PTE	4
			28	F	MAND	1° HPT	PTE	3
			55	F	MAND	1° HPT	PTE	3
			57	F	MAND	1° HPT	PTE	4
			41	F	MAND	1° HPT	PTE	3
			45	F	MAND	1° HPT	PTE	2
			68	F	MAND	1° HPT	PTE	2
			45	F	MAND	1° HPT	PTE	5
			41	F	MAND	1° HPT	PTE	2
			67	F	MAND	1° HPT	PTE	2
			53	F	MAND	1° HPT	PTE	4
			35	F	MAND	1° HPT	PTE	2
			42	F	MAND	1° HPT	PTE	3
			54	F	MAND	1° HPT	PTE	3
			36	F	MAND	1° HPT	PTE	5
			70	F	MAND	1° HPT	PTE	4

Praveen and Thriveni [50]	2012	India	21	F	MAX & MAND	2° HPT	RECON BAR	6 × 7; 2 × 2; 4 × 2

Arunkumar et al. [51]	2012	India	12	F	MAND	2° HPT	EX BT + MED MGMT	na

Soundarya et al. [52]	2011	India	60	M	MAX	1° HPT	EX BT	na

Jakubowski et al. [8]	2011	USA	49	M	MAND	2° HPT	na	8 × 2 × 4

Sutbeyaz et al. [53]	2009	Turkey	53	M	MAX & MAND	1° HPT	PTE	3 × 3; 6 × 7

Proimos et al. [15]	2009	Greece	42	F	MAX	1° HPT	EX BT	2

Rafizadeh et al. [10]	2013	USA	43	M	MAX	1° HPT	na	na

Wilson et al. [12]	2013	USA	26	F	MAX	1° HPT	ICT	na

Mantar et al. [54]	2012	Turkey	23	M	MAX	1° HPT	PTE	na

Nabi et al. [55]	2010	Saudi Arabia	24	F	MAX	2° HPT	PTE	na

Di Daniele et al. [21]	2009	Italy	40	F	MAX	2° HPT	PTE	4

Leal et al. [24]	2006	Brazil	31	F	MAX	2° HPT	PTE + EX BT	na

Oh et al. [9]	2006	USA	53	F	MAX	1° HPT	PTE + EX BT	0.8

Pechalova and Poriazova [17]	2013	Bulgaria	19	M	MAND	2° HPT	EX BT	5
			49	F	MAX	2° HPT	EX BT	na

Sia et al. [56]	2012	China	29	F	MAX	1° HPT	PTE + EX BT	3.7 × 4.3 × 4.3

Bahrami et al. [57]	2012	Iran	38	F	MAX	1° HPT	PTE + EX BT	na

Walsh et al. [11]	2005	USA	13	F	MAX & MAND	1° HPT	PTE + EX BT	na

Di Fede et al. [58]	2013	Italy	71	M	MAND	1° HPT	na	na

Alhusban and Baqain [59]	2011	Jordan	45	F	MAND	1° HPT	PTE + EX BT	na

Pahlavan and Severin [60]	2006	Germany	21	M	MAND	1° HPT	PTE	na

Altay et al. [61]	2013	Turkey	59	M	MAX	3° HPT	PTE	3.8 × 4.6 × 6.8

Artul et al. [62]	2013	Israel	46	F	MAX	2° HPT	MED MGMT	2.2

Guldfred et al. [63]	2012	Denmark	34	F	MAX	1° HPT	EX BT	na

Nair et al. [25]	2011	India	35	F	MAND	2° HPT	MED MGMT	2.7 × 2.5 × 1.9

Magalhães et al. [64]	2010	Brazil	58	F	MAX & MAND	3° HPT	PTE	na

Pati et al. [65]	2014	India	34	M	MAX	1° HPT	na	5.4 × 5.9 × 5.2

Mori et al. [66]	2013	Japan	52	F	MAX	1° HPT	PTE	na

Benjelloun et al. [67]	2007	Morocco	17	F	MAX	2° HPT	PTE	na

Thomas et al. [68]	2011	India	27	F	MAND	2° HPT	EX BT + MED MGMT	4 × 6

Guerrouani et al. [69]	2013	Morocco	41	F	MAX	1° HPT	PTE	na

Mandible = MAND, maxilla = MAX, hyperparathyroidism = HPT, not available = na, male = M, female = F, parathyroidectomy = PTE, excision of brown tumor = EX BT, curettage of brown tumor = CUR BT, medical management = MED MGMT, intralesional corticosteroid and calcitonin therapy = ICCT, mandibulectomy and reconstruction bar placement = RECON BAR, and intralesional corticosteroid therapy = ICT.
